# Advances in the application of artificial intelligence-driven multi-modal imaging technologies in the comprehensive diagnosis and treatment of diabetic foot ulcers

**DOI:** 10.1007/s11154-026-10041-w

**Published:** 2026-05-13

**Authors:** Lianbo Yang, Yifan Wang, Tianqi Wang, Jiahui Zhou, Jinghang Lv, Haidong Liang

**Affiliations:** 1 Center for Wound Repair and Regenerative Medicine, The Second Hospital of Dalian Medical University, No. 467 Zhongshan Road, Liaoning 116000 dalian, China; 2 Department of Orthopaedics, The People’s Hospital of Liaoning Province, No. 33, Wenyi Road, Shenyang, Liaoning 110000 China; 3 Dalian Medical University, No. 9 West Section of Lvshun South Road, Dalian, Liaoning 116044 China

**Keywords:** Artificial intelligence, Diabetic foot ulcer, Multi-modal imaging, Diagnosis and treatment workflow, Radiomics

## Abstract

Diabetic foot ulcers are serious skin wounds that affect many people with diabetes, often leading to severe infections or even the loss of a limb. This paper explores how artificial intelligence —computer programs that can learn from data—is changing the way doctors find and treat these wounds. By reviewing 68 recent studies, we looked at how these smart technologies analyze different types of medical images to help patients. Our findings show that AI can help doctors identify health risks much earlier than traditional methods. These computer tools are also excellent at measuring how a wound is healing and predicting which treatments will work best for each individual. Because AI can spot tiny patterns in images that the human eye might miss, it makes medical care more precise and consistent. In conclusion, using AI to manage diabetic foot wounds offers a powerful way to improve patient health. By helping doctors make better, data-driven decisions, this technology can lead to faster healing and reduce the risk of serious complications for people living with diabetes.

## Introduction

Diabetes has become a global health crisis of unprecedented proportions. According to the latest IDF Diabetes Atlas (11th Edition, 2025), it is estimated that 588.7 million adults (aged 20–79) are currently living with diabetes worldwide. This number is projected to escalate to 852.5 million by 2050, representing a 45% increase. The economic burden is equally staggering, with global diabetes-related health expenditure exceeding one trillion USD in 2024 [1]. Diabetic foot, as one of its most severe complications, has imposed a substantial burden on global healthcare systems. DFU, the core clinical manifestation of diabetic foot, affects approximately 18.6 million patients annually [2] and is associated with an extremely poor prognosis: the lifetime risk of ulcer development for patients reaches 19%–34%, the recurrence rate is as high as 65%, the lifetime amputation risk is approximately 20%, and the 5-year mortality rate escalates to 50%–70%, with about 10% of patients dying within one year of their initial DFU diagnosis [3]. DFU not only severely compromises patients’ quality of life but also results in significant consumption of medical resources due to high treatment costs and disability rates.

The traditional DFU diagnosis and treatment model faces significant limitations across the complete workflow of “Prevention - Diagnosis - Assessment - Prognosis”: early risk screening relies heavily on clinical experience and single indicators, making it difficult to achieve large-scale, low-cost, precise early warnings; the diagnostic phase, while dependent on imaging modalities such as X-ray, Computed Tomography (CT), and Magnetic Resonance Imaging (MRI), suffers from issues including insufficient sensitivity (e.g., the weak capability of X-ray in identifying early infections [4], strong subjective interpretation (e.g., MRI interpretation depends heavily on physician experience [5], and poor accessibility (e.g., the high cost of MRI); wound assessment predominantly utilizes manual description and measurement, lacking objective and quantitative standards; and prognostic prediction relies on population-based statistical regularities, failing to achieve individualized risk stratification. These limitations lead to core pain points in DFU management, characterized by “delayed intervention, assessment bias, and ambiguous prognosis,” urgently necessitating technological innovation to overcome these bottlenecks.

Facilitating clinical translation requires comprehending Artificial Intelligence not merely as a black box but as a computational architecture designed to codify clinical expertise through iterative pattern recognition. Analogous to the accumulation of clinical intuition via longitudinal observation, Deep Learning employs layered artificial neuron networks to perform hierarchical feature extraction, processing low-level morphological details from high-dimensional datasets into complex pathognomonic patterns such as necrotic tissue or hyperkeratosis. This optimization process iteratively adjusts internal model parameters to minimize diagnostic variance against gold-standard labels, transforming multi-modal medical data into actionable intelligence by uncovering latent correlations often elusive to conventional statistical methods.

The rapid development of Artificial Intelligence (AI) technologies, particularly Machine Learning (ML) and Deep Learning (DL), has provided a critical pathway for the revolution of DFU diagnosis and treatment [6–8]. AI can automatically mine deep patterns from multi-modal data (including clinical indicators, medical images, and biomechanical signals), facilitating a transformation from “experience-driven” to “data-driven” paradigms: in the early warning stage, ML models based on clinical biochemical data can achieve efficient screening of high-risk DFU populations; in the diagnostic phase, DL models can accurately interpret complex medical images, enabling the objective differentiation of challenging-to-diagnose conditions such as Osteomyelitis (OM) and Charcot Neuro-osteoarthropathy (CNO); in the wound assessment stage, Semantic Segmentation models can quantify wound size and tissue composition; and in the prognostic stage, ML models integrating multiple features can predict risks of healing, amputation, and mortality.Given the technical complexity and the imperative for rigorous clinical integration, this review systematically explores the current landscape, technical benchmarks, and future trajectory of AI-driven strategies to provide a comprehensive roadmap for researchers and practitioners in diabetic foot management.

## Methodology

This systematic review covers machine learning (ML) and deep learning (DL) applications in diabetic foot ulcer (DFU) clinical pathways. The mixed‑methods framework follows PRISMA guidelines [9] and SLR methodological recommendations [10]. The review process has four core stages:

Research questions and objectives.

Research scope.

Literature search and screening.

Quality appraisal and clinical pathway mapping.

### Research questions and objectives

This review maps current ML/DL applications in diabetic foot management and their future directions. The primary question is how these techniques perform across the DFU lifecycle—early warning, diagnosis, assessment, and prognosis.

Specific objectives:

Systematically review ML/DL use in DFU and pinpoint technical entry points at each clinical stage.

Classify ML/DL technologies by clinical pathway functions: risk prediction, lesion detection, differential diagnosis, wound assessment, and prognosis.

Assess methodological quality with predefined criteria to ensure analytical robustness.

Identify clinical translation challenges: interpretability, generalizability, and vascular intervention limitations.

### Research scope

This review targets diabetic foot management and is restricted to ML and DL. The framework focuses on integrating ML/DL into DFU clinical decision support systems, with an emphasis on multimodal image fusion.

### Literature search and screening

Databases and search strategy.

We searched PubMed, Web of Science Core Collection, and IEEE Xplore. To specifically capture ML/DL studies, the search queries used only “Machine Learning”, “Deep Learning”, and their abbreviations (ML, DL); the broader term “Artificial Intelligence” was excluded. Search strategies were iteratively refined and are shown below:

PubMed (MeSH included):

text.

((“Machine Learning“[Mesh] OR “Deep Learning“[Mesh] OR “Machine Learning“[Title/Abstract] OR “Deep Learning“[Title/Abstract] OR “ML“[Title/Abstract] OR “DL“[Title/Abstract])) AND ((“Diabetic Foot“[Mesh] OR “Diabetic Foot Ulcer“[Title/Abstract] OR “DFU“[Title/Abstract]))

Web of Science Core Collection (topic search TS):

text.

TS=((“machine learning” OR “deep learning” OR “ML” OR “DL”)) AND TS=((“diabetic foot” OR “diabetic foot ulcer” OR “DFU”)).

IEEE Xplore (full‑text & metadata):

text.

((“All Metadata”:“machine learning” OR “All Metadata”:“deep learning” OR “All Metadata”:ML OR “All Metadata”:DL)) AND ((“All Metadata”:“diabetic foot” OR “All Metadata”:“diabetic foot ulcer” OR “All Metadata”:DFU)).

The search covered database inception to November 2025 and was limited to peer‑reviewed original research articles and full‑length conference papers in English.

Search results.

The initial search returned 1057 records:PubMed 285 Web of Science 473, IEEE Xplore 299. After import into EndNote and deduplication, 410 unique records proceeded to screening.

Inclusion and exclusion criteria.

Inclusion:

1. Studies applying ML or DL to DFU risk prediction, diagnosis, grading, wound assessment, or prognosis.
2. Original research or full conference papers.
3. English language.

Exclusion:

1. Non‑ML/DL AI methods only (e.g., expert systems, fuzzy logic).
2. Healthy‑participant‑only studies without DFU patient validation.
3. No original data or model performance metrics reported.
4. Reviews, editorials, case reports, commentaries, or other non‑original work.
5. Non‑English publications.

Screening process.

Two reviewers independently screened all records; disagreements were settled through discussion or third‑party arbitration.

Title/abstract screening: 294 records were excluded, leaving 116 articles for full‑text retrieval.

Full‑text retrieval: Full texts of 108 articles were obtained; 8 articles could not be retrieved.

Full‑text review: Applying the same criteria, 40 articles were excluded: not focused on AI/ML/DL application (*n* = 15), review/editorial (*n* = 12), insufficient data (*n* = 8), duplicate population (*n* = 5).

Reference list screening: Manual checking of included study bibliographies identified no additional eligible studies.

Final inclusion.

**Fig. 1:**
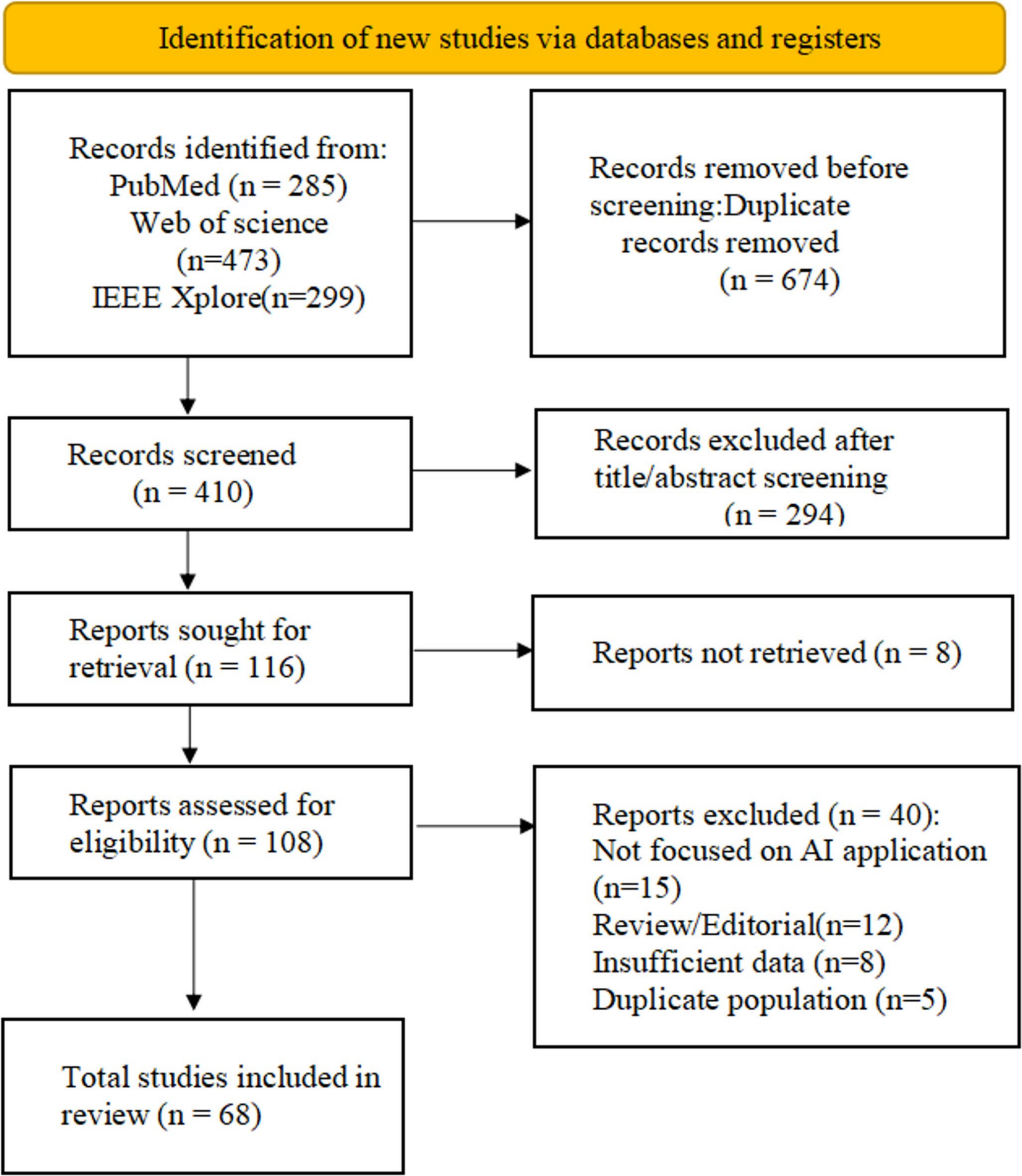
Flowchart of literature search and selection process

68 studies directly relevant to DFU clinical pathways were ultimately included. The PRISMA flow diagram is provided in Fig. 1.

## Results

**Fig. 2:**
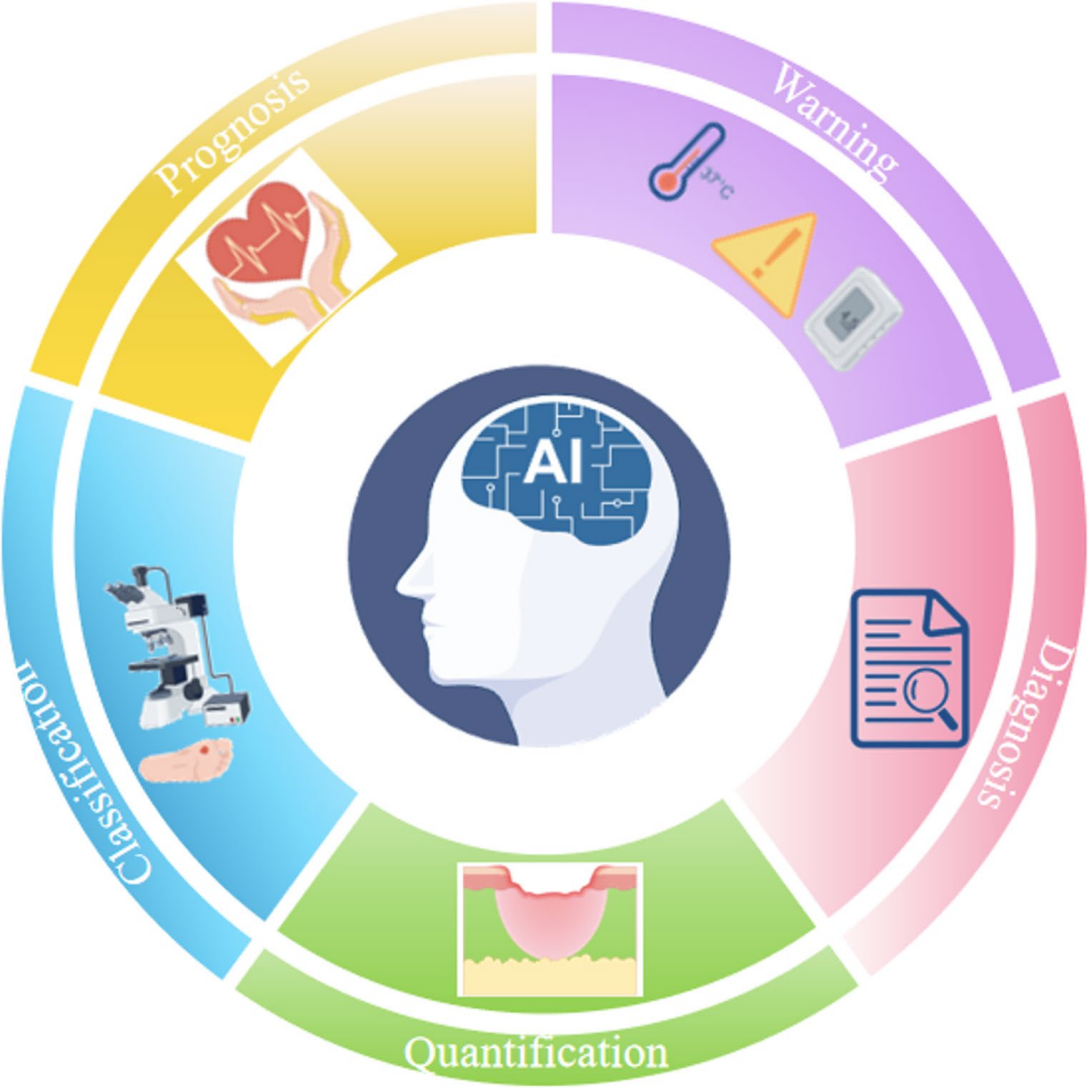
Key stages in the clinical pathway of diabetic foot ulcer management where AI technologies are applied

The 68 included studies were categorised according to the DFU clinical pathway stages illustrated in Fig. 2: early risk warning (*n* = 19), diagnosis and differential diagnosis (*n* = 5), wound quantification and segmentation (*n* = 8), grading and classification (*n* = 16), and prognostic prediction (*n* = 20).

**Fig. 3:**
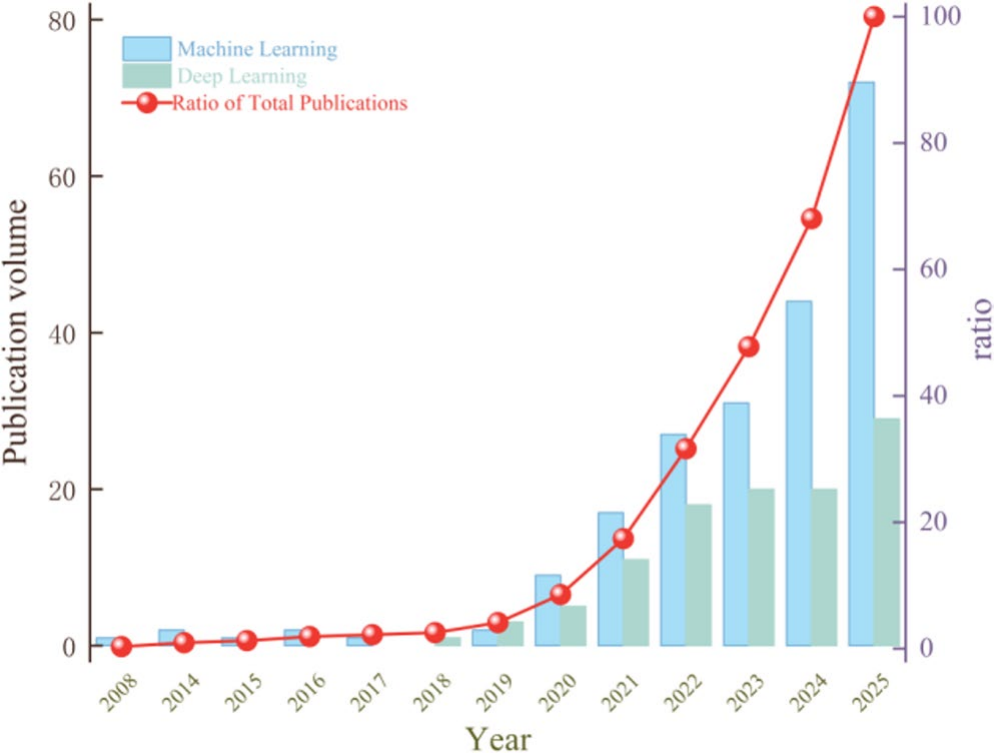
Annual number of publications on artificial intelligence in diabetic foot ulcers

Publication trends are shown in Fig. 3. Annual publication output on ML/DL in DFU has risen sharply since 2021. Research focus has shifted from single‑task image classification to multimodal data fusion and explainable AI, paralleling the global increase in DFU prevalence and the growing demand for precision medicine.

## AI-driven early identification and risk warning

The application of AI technologies is fundamentally shifting the management strategy for DFU from a reactive model of “treating established disease” to a proactive paradigm focused on “preventing disease onset.” Through the in-depth analysis of multi-source data, AI enables a prospective and individualized assessment of ulceration risk.

### Risk prediction models based on clinical and biochemical data

Utilizing routinely accessible clinical and biochemical data to construct ML prediction models is one of the most effective pathways for realizing large-scale, low-cost screening for DFU. Xiaoling, W. [11] et al. employed an automated machine learning (AutoML) ensemble framework, leveraging 50 risk factors (including 9 local foot examination metrics) from 566 patients, to construct a DFU risk prediction model. The model achieved an Area Under the Curve (AUC) of 88.48% in the training set and exhibited stable performance in external dataset validation (AUC = 0.762). This model not only validated the significance of traditional risk factors, such as peripheral neuropathy symptoms, but also identified non-traditional risk factors, including varicose veins of the lower extremities and a history of cerebral infarction. This provides a new perspective for research into the pathophysiological mechanisms of DFU. Similarly, Wang, Y. [12] et al. focused on a high-risk population of patients with type 2 diabetes complicated by lower extremity arteriosclerosis obliterans (ASO). Based on clinical and laboratory data from 1,978 patients, they utilized multiple algorithms, such as Logistic Regression and LASSO(Least Absolute Shrinkage and Selection Operator)—a regression-based method that applies an L1 penalty to drive weak feature coefficients to zero, thereby performing simultaneous feature selection and regularization—to develop a DFU prediction model comprising 10 independent risk factors. The model demonstrated extremely high predictive accuracy across the training, internal validation, and external temporal validation sets (highest AUC up to 0.977), and its visual nomogram tool significantly enhanced its clinical utility. The widespread success of such data-driven studies further corroborates the core value of ML in DFU risk prediction: Shi, G. [13] et al. using a Multi-Layer Perceptron (MLP) model, precisely identified individuals at high risk for neuropathic foot ulcers (NFU) from a cohort of patients with diabetic peripheral neuropathy (DPN), achieving an AUC of 0.901; an XGBoost model by Jian, Y. [14] achieved the simultaneous prediction of eight diabetic complications, including DFU, attaining a DFU prediction accuracy of 97.7%; and a study by Wu, Y. [15] et al. focused on DPN and lower extremity arterial disease (LEAD), two critical precursor conditions for DFU. While identifying their specific risk factors, the study emphasized the significance of addressing common risk factors—such as the urinary albumin/creatinine ratio and glycated hemoglobin—in reducing the overall risk of DFU. Furthermore, Ferreira, A. [16] et al. demonstrated that by using only patient-reported questionnaire data—without any diagnostic labels—unsupervised machine learning methods, which identify hidden patterns in unlabeled data through clustering or density estimation, can screen for individuals at high risk for Diabetic Foot (DF) with high precision (90% accuracy). This contrasts with supervised approaches, which require labeled outcomes (e.g., ulcer vs. non-ulcer) to train predictive models. Their findings provide an alternative and effective pathway for low-cost preliminary screening at the community level.

### Early detection based on physiological functional signals

When the sensitivity of clinical data reaches a bottleneck—i.e., when predictive performance plateaus due to the inherent insensitivity of conventional indicators to subclinical pathology— AI shifts to the direct analysis of early physiological functional changes associated with DFU precursor lesions, providing more objective and precise screening tools for clinical practice.

#### Plantar pressure and biomechanical analysis

DPN leads to abnormal biomechanical distributions in the plantar region, a characteristic that has become a core analytical target for AI models. Sheikh, M.M. [17] et al. utilizing plantar pressure images from 86 patients with varying degrees of neuropathy, developed an automatic image segmentation algorithm to precisely delineate the forefoot and rearfoot regions. Upon comparing multiple ML models, they found that static plantar pressure analysis outperformed dynamic analysis. Specifically, their gradient boosting model achieved 100% accuracy in DPU prediction, enabling the ultra-early quantitative diagnosis of neuropathy. Aman, A. [18] et al. further advanced this by fusing plantar pressure and temperature signals, constructing a low-complexity ML pipeline. This system not only achieved 99.58% accuracy in blind testing but was also optimized for low-resource hardware (81.2 kB memory usage, 1.31 s latency), laying a foundation for home-monitoring scenarios. Additionally, Haque, F. [19] et al. analyzed electromyography (EMG) and ground reaction force (GRF) data during the gait cycle. By using a feature-optimized k-Nearest Neighbors (KNN) model—which classifies patients by comparing their data to the most similar historical cases—they achieved 98.68% accuracy in stratifying DPN severity.

The clinical utility of these biomechanical markers is best exemplified by the work of Chauhan, A.S. [20] et al. In conventional settings, diagnosing DN often involves reactive testing (e.g., monofilament or vibration perception) once sensory loss has already progressed. Chauhan et al. demonstrated that supervised machine learning methods—which learn to map complex biological inputs to expert-defined diagnostic labels—can identify the earliest biomechanical shifts. By combining plantar pressure features with clinical metadata, their model differentiated among pre-diabetes, diabetes without neuropathy, and diabetes with peripheral neuropathy with 94–100% precision. In this study, neuropathy status was assigned according to the authors’ clinical diagnostic labels in the source dataset (as the reference standard), and future work should report the confirmatory criteria used (e.g., monofilament, vibration perception threshold/biothesiometry, or nerve conduction studies) to enhance reproducibility and facilitate external validation.This creates a proactive diagnostic window, allowing clinicians to utilize AI as a “biomechanical auxiliary tool” to detect nerve dysfunction at its subclinical inception, thereby preventing the transition from early neuropathy to limb-threatening ulceration. Accordingly, intermediate endpoints (e.g., plantar pressure reduction) should be explicitly linked to downstream clinical outcomes rather than treated as sufficient surrogates; prospective validation is still needed to confirm endpoint-level benefits (e.g., reduced incident ulceration/re-ulceration and improved time-to-heal).

#### Thermal imaging and microcirculatory function analysis

Thermal imaging technology, by capturing abnormal foot temperature distributions caused by autonomic neuropathy, has become an auxiliary tool for assessing DF risk. AI models can quantify this abnormal pattern, thereby enabling objective screening [21–23]. Autonomic neuropathy affects cutaneous blood flow and sweat gland function, leading to abnormal foot temperature distributions, while alterations in microcirculatory parameters also provide a new dimension for DPN screening. A comparative study by Khandakar A. [24] et al. demonstrated that an AdaBoost model, optimized with the top-10 features selected via Random Forest, achieved an F1 score of 96.70% in early detection using foot thermal images. Its performance surpassed that of some DL models (e.g., MobileNetV2 at 95.82%), highlighting the application value of traditional ML in specific scenarios. Furthermore, this model possesses the potential for smartphone deployment. Zhang, X. [25] et al. explored an alternative approach, measuring microcirculatory parameters using Laser Doppler and transcutaneous oxygen tension techniques. The Random Forest model, constructed based on a post-occlusive reactive hyperemia (PORH) dataset, achieved 84.6% accuracy and an AUC of 0.894, establishing it as a reliable screening tool for DPN. This work opens a new functional perspective for early detection.

#### Early etiological assessment based on complex medical imaging

For more complex medical imaging, DL demonstrates its unique advantages. Wu, C. [26] et al. improved the YOLOv5 model to achieve automatic detection and grading of stenotic lesions in lower extremity CT Angiography (CTA) images, effectively overcoming interference from calcification. This provides a rapid and objective tool for the vascular etiological assessment of DFU. A multi-modal DL model constructed by Tian, Z. [27] et al. innovatively fused tongue images with clinical data. It not only achieved high performance but also confirmed the unique value of objectified Traditional Chinese Medicine (TCM) tongue diagnostics in DF risk prediction. Meanwhile, a multi-center study conducted by Li, Y. [28] et al. utilized stacked ensemble learning combined with extensive clinical data, demonstrating that a multi-model system integrating ML and DL can provide stable and accurate DF risk assessment. Hernandez-Guedes, A. [29] et al. employed DL-based variational methods to extract a novel feature set from thermal images. The resulting classification performance significantly surpassed the levels of previous research, showcasing the potential of DL in feature discovery (Table 1).

**Table 1:** Performance of AI models in early identification and risk prediction of diabetic foot ulcers

Category	Author	Technology	Core Model	Data Scale and Type	Core Performance Results
Clinical Data	Xiaoling, W. [11] et al.	Machine Learning	AutoML	566 patients, 50 clinical/biochemical indicators	AUC: 88.48%; Accuracy: 87.33% (External validation AUC: 0.762)
	Wang, Y. [12] et al.	Machine Learning	Logistic, LASSO, SVM, RF	1978 ASO diabetic patients clinical data	Training set AUC = 0.962; External validation AUC = 0.977
	Shi, G. [13] et al.	Machine Learning	MLP	400 DPN patients clinical data	Accuracy: 0.875; AUC: 0.901
	Jian, Y. [14]	Machine Learning	XGBoost	884 patient records, 79 features	Diabetic foot accuracy: 97.7%; F1: 97.8%
	Wu, Y. [15] et al.	Machine Learning	XGBoost, RF	479 diabetic patients clinical data	DPN diagnosis (XGBoost): F1 = 83.7%; LEAD diagnosis (RF): F1 = 82.8%
	Ferreira, A. [16] et al.	Machine Learning	Unsupervised Learning	250 DM patients questionnaire data	Accuracy: 90%; Specificity: 100%
Imaging & Signal	Sheikh, M.M. [17] et al.	Machine Learning	Gradient Boosting	86 patients, 172 plantar pressure maps	Static analysis accuracy: 100%
	Aman, A. [18] et al.	Machine Learning	k-NN	43 subjects plantar pressure/temperature signals	Accuracy: 99.58%; AUC: 99.56%
	Haque, F. [19] et al.	Machine Learning	KNN	21 subjects EMG/GRF signals	Accuracy: 98.68% (based on GRF analysis)
	Khandakar A. [24] et al.	Machine Learning	AdaBoost	167 foot thermal images	F1-score: 96.70% (outperformed DL models)
	Zhang, X. [25] et al.	Machine Learning	Random Forest	261 subjects microcirculation parameters	Accuracy: 84.6%; AUC: 0.894
	Wu, C. [26] et al.	Deep Learning	YOLOv5	460 CTA images	Stenosis grading detection mAP significantly superior to control algorithm
	Tian, Z. [27] et al.	Deep Learning	ResNet-50	391 subjects tongue images + clinical data	Accuracy: 0.95; F1-score: 0.9392
	Li, Y. [28] et al.	Deep Learning	Stacking (LR)	6180 patients clinical data	Test set AUC: 0.938; Accuracy: 0.877
	Hernandez-Guedes, A. [29] et al.	Deep Learning	SVM (DL features)	244 plantar thermal images	F1-score: 90.27% (based on features selected by DL method)

## Intelligent diagnosis and differentiation: improving the objectivity and consistency of diagnosis

In the diagnostic phase, the core advantage of AI technology lies in its ability to codify the diagnostic expertise and logical reasoning of expert clinicians into standardized algorithms. This methodology effectively overcomes the limitations of conventional diagnosis, which is often reliant on subjective experience and suffers from a lack of consistency. AI provides objective, reproducible auxiliary support for the differentiation of complex complications, disease staging, and status assessment. This capability is of substantial significance for optimizing treatment decisions and improving patient prognosis.

### Precise staging of complications

Diabetes mellitus and its associated foot complications represent a continuous developmental process. Consequently, the precise differentiation of various stages is critical for therapeutic decision-making. Pan, Y. [30] et al. utilized a neural network optimized by a genetic algorithm to successfully construct a model capable of distinguishing among “uncomplicated diabetes mellitus,” “diabetes with peripheral vascular disease (DPVD),” and “DF.” The core value of this research lies in its provision of a reliable quantitative computational tool to address the problem of precise staging within the continuous spectrum of diabetic complications. This approach helps to avoid the issues of under-treatment or over-treatment that can result from generalized diagnoses.

### Intelligent interpretation of medical imaging

DL has demonstrated unique advantages in feature extraction and pattern recognition from high-dimensional, complex medical images. It has subsequently become the core technological support for the intelligent interpretation of DF and DFU related imaging. Applications are primarily concentrated on three major directions: the differentiation of core diseases, the classification of wound types, and the assessment of ulcer status.

#### Precise differentiation of core diseases

The differentiation between Osteomyelitis (OM) and Charcot Neuro-osteoarthropathy (CNO) represents a critical diagnostic crossroad in diabetic foot care. While CNO is fundamentally a clinical diagnosis—integrating patient history, inflammatory markers, and physical findings—its radiographic presentation frequently mimics that of OM, leading to a high risk of misdiagnosis. Moreover, although the simultaneous occurrence of OM and CNO is clinically rare, the inability to distinguish them can result in diametrically opposed and potentially catastrophic treatment paths (e.g., unnecessary surgical debridement versus specialized offloading). Cakir, M. [31] et al. addressed this “diagnostic gray zone” by developing a DL model based on MRI. This model does not replace the physician’s clinical judgment but rather serves as a high-precision objective auxiliary tool. By achieving a differentiation accuracy exceeding 95%, the AI captures subtle radiographic texture signatures—such as specific marrow edema patterns or periosteal reactions—that may be imperceptible to the human eye.

#### Multi-type classification of chronic wounds

Busch, D.A. [32] et al. demonstrated the capability of AI in broader-spectrum disease differentiation. The ConvNeXt model they employed achieved a 90% unbalanced accuracy on a dataset comprising five types of chronic leg ulcers (totaling 3,674 photographs). The model exhibited high sensitivities for the identification of pyoderma gangrenosum and DFU, reaching 94% and 97%, respectively. This work lays a solid foundation for future clinical decision support systems.

#### Identification of key DFU states

The work by Almufadi, N. [33] et al. focused specifically on DFU. They employed a strategy combining a pre-trained Convolutional Neural Network (CNN) with a customized head model and a ML classifier. This approach achieved accuracies of 97% and 93% in the DFU image classification tasks for ischemia and infection, respectively. This study demonstrated the effectiveness of the proposed method in identifying critical DFU states (Table 2).

**Table 2:** Performance of AI models in the differential diagnosis and staging of diabetic foot complications

Author	Technology	Core Model	Data Scale and Type	Core Performance Results
Pan, Y. [30] et al.	Machine Learning	GA-BPNN	1240 patients clinical data	Task G2 (differentiating DPVD from DF) AUC: 0.89; Accuracy: 0.83
Cakir, M. [31] et al.	Deep Learning	ResNet, EfficientNet	148 patients MRI data	Accuracy > 95%; Sensitivity > 92%; Specificity > 97%
Almufadi, N. [33] et al.	Deep Learning	EfficientNetB0	DFU image dataset	Ischemia classification accuracy: 97%; Infection classification accuracy: 93%

## Objective wound quantification and precise segmentation

Following the onset of a DFU, the precise and objective assessment of wound size, morphological parameters, and tissue composition serves as the core prerequisite for formulating individualized treatment plans, dynamically adjusting intervention strategies, and scientifically evaluating therapeutic efficacy. Traditional wound assessment, which relies on manual descriptions and measurements, suffers from significant limitations, including strong subjectivity, poor inter-rater consistency, and insufficient quantitative precision. The development of AI technologies, especially DL semantic segmentation models, has provided crucial technological support for the standardized management of DFU.

### Semantic segmentation-driven fine-grained wound assessment

Semantic segmentation models achieve pixel-level semantic understanding of images through an encoder-decoder architecture. They can precisely delineate the wound area from normal tissue and differentiate various tissue components within the wound bed, serving as the core technology for fine-grained wound assessment. The SeeWound 2 tool, developed by Lindborg, K. [34] et al., provides a benchmark for such validation. In their study, the AI’s performance was compared against expert manual planimetry and 3D laser scanners (the current gold standards). The tool demonstrated high concordance with these clinical benchmarks, achieving 96.28% accuracy for area and 90.75% for depth. This robust validation ensures that the morphological parameters captured by smartphone-integrated LiDAR sensors are as reliable as traditional, more labor-intensive clinical measurements.

Further advancing this field, Zhou, G. X. [35] utilized models like Mask2former to segment six complex tissue types (granulation, necrotic tissue, bone, etc.). The “gold standard” here involved multi-expert consensus labeling, where the AI’s tissue identification was cross-referenced with clinical diagnoses of infection and ischemia. The profound clinical significance of this fine-grained segmentation lies in its ability to define a “Healing Trajectory” as a powerful clinical endpoint.

In clinical wound research, a healed wound is defined by complete re-epithelialization. AI-quantifiable pre-healing signatures redefine this endpoint. A successful healing trajectory is characterized by a decreasing slough percentage and an increasing ratio of healthy granulation tissue. Furthermore, the AI-calculated four-week area reduction rate predicts twelve-week complete healing.AI-driven Granulation/Necrosis Ratio continuous objective biomarkers reflecting wound bed micrometabolic status offer more sensitive clinical trial endpoints than binary healed/unhealed outcomes from the subjective Wagner scale. Manual description to AI-quantified metrics transition standardizes wound assessment into a trackable clinical procedure directly addressing inter-observer variability in DFU management.

### Diverse technical solutions and data infrastructure support

The rapid development in the field of DFU wound quantification and segmentation is attributable to both the innovative exploration of diverse technological pathways and the comprehensive construction of high-quality data resources. This synergy has established a virtuous cycle of “technology-driven breakthroughs and data-driven support.”

At the level of technological exploration, P, J. [36] et al. proposed the AFSegGAN model. This model which utilizes a U-Net as the generator and aPatch discriminator as its core, was trained on the MICCAI 2021 Foot Segmentation Challenge dataset (expanded to 4,040 images via data augmentation). It achieved precise DFU segmentation, attaining a Dice coefficient of 93.11% and an Intersection over Union (IoU) of 99.07%. Its performance surpassed the top-performing methods from the contemporary challenge, thereby demonstrating the advantages of conditional Generative Adversarial Networks (cGANs) in medical image segmentation. Concurrently, the DFUCare platform developed by Sendilraj, V. [37] et al. realized technological integration. It employs YOLOv5 for wound localization, while utilizing InceptionResNetV2 and DenseNet121 for the classification of infection and ischemia, respectively. By combining image preprocessing with feature analysis, the platform establishes a one-stop analysis workflow—encompassing DFU localization, classification, and size measurement—which has demonstrated significant potential for telemedicine applications in clinical validation. In earlier research, Wang, L. [38] et al. proposed a cascaded two-stage Support Vector Machine (SVM) classifier combined with a Conditional Random Field (CRF) refinement technique. This approach achieved an average sensitivity of 73.3% and an average specificity of 94.6% on 100 DFU images, confirming the feasibility of traditional ML in wound segmentation. Furthermore, Huang, H.N. [39] et al. employed a Fast R-CNN model optimized by transfer learning, integrated with GrabCut segmentation and SURF scale calibration techniques. This method achieved a 90% wound recognition accuracy on 727 DFU images (augmented to 3,600), offering a valuable reference for the integrated application of DL and conventional image processing technologies.

Regarding the construction of data infrastructure, Costa, T. [40] et al. utilized traditional image processing techniques (e.g., HSV color space segmentation, Canny edge detection) to automatically localize the nine standard sites for the Semmes-Weinstein Monofilament Examination (SWME) in 90 plantar images from 15 healthy volunteers. The localization accuracy exceeded 85% for seven of these sites. This work established a preliminary foundation for the automated physical examination and multi-modal assessment of DFU. Addressing the challenge of insufficient standardized datasets in DFU research, Basiri, R. [41] et al. proposed a standardized acquisition protocol for multi-modal images (RGB, thermal, and depth maps) and clinical metadata. They constructed the Zivot dataset, which includes approximately 3,700 sets of multi-modal images from 269 patients (notably containing comparative images from before and after debridement). A UNet baseline model trained on this dataset (utilizing EfficientNet as the feature extractor) achieved excellent performance, with an F1 score of 0.79 and a mean Average Precision (mAP) of 0.86. This contribution provides a standardized data framework and an invaluable resource for subsequent multi-modal DFU analysis and DL research (Table 3).

**Table 3:** Performance of various ai technologies in diabetic foot ulcer wound segmentation and assessment

Author	Technology	Core Model	Data Scale and Type	Core Performance Results
Lindborg, K. [34] et al.	Deep Learning	U-Net ×2	3383 wound images + LiDAR	Surface area accuracy: 96.28%; Depth accuracy: 90.75%
Zhou, G. X. [35] et al.	Deep Learning	Mask2former	671 DFU images	Best overall segmentation performance (IoU, Dice)
P, J. [36] et al.	Deep Learning	AFSegGAN (cGAN)	1010 training images	Dice score: 93.11%; IoU: 99.07%
Sendilraj, V. [37] et al.	Deep Learning	YOLOv5, InceptionResNetV2	DFUC2020/2021 dataset	Wound size measurement error: ±0.2–0.3 cm
Wang, L. [38] et al.	Machine Learning	Cascaded SVM	100 foot ulcer images	Average sensitivity: 73.3%; Average specificity: 94.6%
Huang, H.N. [39] et al.	Deep Learning	Fast R-CNN	727 wound images	Wound recognition accuracy: 90%
Costa, T. [40] et al.	Machine Learning	Traditional Image Processing	90 plantar color images	Localization accuracy > 85% for 7 of 9 sites
Basiri, R. [41] et al.	Deep Learning	U-Net	3700 sets of multi-modal images from 269 patients	F1-score: 0.79; mAP: 0.86 (baseline model performance)

## Classification and prognosis: from population statistics to individualized insight

In the clinical management of DFU, precise severity classification and individualized prognostic prediction are critical links for optimizing therapeutic strategies and improving patient outcomes. Traditional classification and prognostic assessment often rely on population-based statistical regularities and the subjective experience of clinicians, suffering from limitations such as poor consistency and insufficient specificity. AI technology, leveraging its capabilities in multi-dimensional information fusion and complex pattern recognition, breaks the constraints of traditional assessment frameworks. It achieves a leap from “population-based standards” to “individual-level precision,” providing objective and reliable technological support for the individualized classification, risk stratification, and prognostic judgment of DFU.

### Grading and classification of diabetic foot

Classification models based on AI are progressively establishing more objective, consistent, and reproducible systems for DFU grading and classification by learning the latent features from multi-source data, including images and three-dimensional morphology. This development provides standardized tools for clinical assessment. Beyond image data, ML can also classify DFU based on clinical metadata. Basiri, R. [42] et al. utilized a Random Forest model, relying solely on clinical wound assessment data, to classify the healing phases of DFU (inflammatory, proliferative, and remodeling) with a mean AUC of 0.73. This work provides an automated tool for medical triage. In the context of this review, “clinical metadata” is explicitly defined as the structured, non-imaging patient information typically extracted from Electronic Health Records (EHRs). This encompasses three core dimensions: demographic variables (e.g., age, gender, duration of diabetes), physiological parameters (e.g., blood pressure, Ankle-Brachial Index), and biochemical indicators (e.g., HbA1c, C-reactive protein, WBC count). Integrating these metadata with imaging data allows AI models to contextualize visual features within the patient’s systemic metabolic status, thereby improving diagnostic specificity.

#### Grading and classification based on visible light images

Owing to their ease of acquisition, visible light images have emerged as the primary data source for AI-driven grading and classification. Research in this domain is concentrated on four principal directions: DFU severity scoring, classification of lesion types, adaptation for three-dimensional (3D) foot morphology, and the enhancement of model interpretability.

Wang, Z. [43] et al. developed ScoreDFUNet, an intelligent DFU scoring system. The system combines a U-Net segmentation model with a ResNet50 classifier. The segmentation module quantifies six lesion-area categories, including ulceration, infection, and gangrene. The classification module identifies four lesion states. The final severity score is computed using a mathematical formula that integrates segmented areas, class probabilities, and predefined weights. The model was trained on 1,944 images and evaluated on 595 external test images from the FUSC 2021 dataset. It achieved a classification accuracy of 95.34%. In physician comparisons, its scoring performance exceeded that of junior and intermediate clinicians and approached senior-level scoring. Collectively, these results support the feasibility of integrating AI-based scoring into clinical decision-support systems for DFU.

Wang, J.G. [44] et al. addressed small sample size and class imbalance in lesion-type classification. They proposed FARRNet, a reconstructed residual network with spatial and channel attention. The model uses an SPCA attention module to enhance feature focus. It also includes a decoder pathway to strengthen feature extraction. For optimization, the authors combined a weighted joint loss (Focal Loss + SSIM Loss) with pseudo-labeling. On the DFUC 2021 dataset, the model achieved a macro-average F1 score of 60.81% and an AUC of 87.37%. The dataset contains four categories: no lesion, infection, ischemia, and mixed infection–ischemia. This study provides a practical strategy for improving DFU classification under small-sample constraints.

In the domain of individualized orthotic device adaptation, Li, P.L. [45] et al. utilized 3D foot scan data from 114 diabetic patients. They employed Principal Component Analysis (PCA) and two-stage clustering to partition foot morphologies into six classes. Subsequently, they constructed a classification model that fuses DiffusionNet geometric deep learning with 13 foot measurement features. This model attained an average accuracy of 82.9%, significantly outperforming baseline models such as Support Vector Machine (SVM) and PointNet++. Crucially, this AI-driven morphological classification may serve as a foundation for personalized offloading strategies aimed at ulcer prevention. By precisely mapping the foot’s unique topology to specific insole geometries, the system may facilitate “total contact” support. This mechanism redistributes peak plantar pressure—a key biomechanical contributor to DFU recurrence—suggesting that AI could help translate static 3D scans into patient-specific orthotic designs. However, evidence that such AI-assisted orthoses consistently reduce DFU incidence or recurrence remains limited, and prospective, endpoint-driven validation is still needed. Therefore, it is essential that intermediate metrics, such as plantar pressure reduction, are tied to definitive clinical outcomes instead of being regarded as self-sufficient surrogate markers.

Regarding classification methodology, Gudivaka, R.K. [46] et al. explored an ‘advanced practical approach to machine learning,’ which integrated multiple techniques such as reinforcement learning and component pattern generation networks. In a six-grade DFU classification task, this approach achieved an overall accuracy of 92.5%, presenting a novel solution for complex classification problems.

Furthermore, the enhancement of model interpretability has become critical for clinical translation. The XAI-FusionNet framework, proposed by Biswas, S. [47] et al., achieves this by parallel-fusing multi-scale features from pre-trained models, including DenseNet201 and VGG19. This framework, combined with three explainable AI (XAI) algorithms (SHAP, LIME, and Grad-CAM), achieved 99.05% accuracy in DFU detection across 6,963 foot images, thereby enhancing the model’s clinical trustworthiness. Similarly, Mahmud, M.I. [48] et al. developed DFU_DIALNet, which utilizes EfficientNetB0 as the feature extractor and an SVM as the classifier. This system integrates Grad-CAM and LIME visualization techniques, achieving over 95% accuracy on a hybrid dataset as well as on two independent public datasets (KDFU and DFUC2020). A corresponding Streamlit web application was also developed, enhancing the technology’s practical utility.

#### Grading and risk stratification based on thermal imaging

Thermal imaging technology, by capturing abnormal foot temperature distributions, offers a unique functional perspective for DFU risk stratification and severity assessment. Research in this area is centered on label standardization, regionalized analysis, model optimization, and methodological selection, progressively enhancing assessment precision and clinical applicability.

To address the core challenges of absent severity labels for thermal images and the unreliability of traditional TCI (Thermal Clinical Index) labels, Khandakar, A. [49] et al. employed an unsupervised learning strategy. Deep features extracted by VGG19 were subjected to dimensionality reduction via Principal Component Analysis (PCA). Subsequently, K-means clustering (k = 3) was applied to reconstruct 167 pairs of foot thermograms into three-tier labels: “mild, moderate, and severe,” which were then validated by medical experts. A model trained on this newly labeled dataset, integrating Adaptive Histogram Equalization (AHE) image enhancement with VGG19, achieved 95.08% accuracy and 97.2% specificity. This work provides a standardized labeling system and validation framework for grading based on thermal imaging.

Sharma, N. [50] et al. further proposed a regionalized analysis strategy. This approach involved segmenting plantar thermal images—assisted by visible light images—and partitioning them into three distinct regions (forefoot, midfoot, and rearfoot) based on ulcer incidence rates and pressure distribution. An InceptionV3 model, trained on “normal, moderate, and severe” three-tier labels, attained an accuracy of 92.7% and an AUC of 0.93. This performance was significantly superior to that of traditional ML methods (e.g., Random Forest accuracy of 83.7%), thereby substantiating the effectiveness of regionalized analysis in enhancing the precision of classification.

In the areas of multi-class classification and customized model optimization, Muralidhara, S. [51] et al., addressing a 6-class classification task (non-diabetic plus 5 TCI grades), proposed a customized CNN model that preserves the original rectangular input dimensions. Combined with offline-online data augmentation techniques, this model achieved a mean accuracy of 98.27%, resolving performance bias issues stemming from class imbalance. A 9-layer CNN model (DFTNet), designed by Cruz-Vega, I. [52] et al., attained a mean accuracy of 94.53% in the TCI five-grade classification task. It demonstrated excellent performance, particularly in differentiating between adjacent grades (Grade 3 vs. Grade 4) with an accuracy of 85.3%, underscoring the advantages of task-specific model design. The hybrid diagnostic framework proposed by Eldin, A.S. [53] et al. fuses ORB handcrafted features with deep features (e.g., from ResNet50), which are then input into a lightweight Deep Neural Network (DNN) classifier. This framework achieved 98.51% accuracy and an AUC of 1.00 on 1,670 thermal images, effectively balancing detection accuracy and real-time processing capabilities.

Multiple studies have validated the feasibility of risk stratification using a combination of thermal imaging and DL: Bagavathiappan, S. [23] et al., based on a ResNet50V2 model, achieved 71.8% accuracy and 81.2% sensitivity for IWGDF risk classification on 153 plantar thermal images; Panamonta, V. [54] et al. employed a ResNet50V2 model optimized with transfer learning to achieve a 71.8% mean accuracy in classifying 124 patients into IWGDF low/high-risk groups, providing a reference for clinical risk screening; Verma, G. [55] et al. preprocessed thermal images using Canny edge detection and watershed segmentation, which, when combined with the EfficientNetB0 model, achieved 99.4% DFU detection accuracy, significantly enhancing model performance.

It is noteworthy that the selection of methodology must be aligned with the specific characteristics of the task: Khandakar, A. [56] et al. found that in a TCI five-grade classification task, a traditional Multi-Layer Perceptron (MLP) model optimized with feature engineering (90.1% accuracy) outperformed the 2D CNN model that was tested, suggesting the advantages of feature engineering in specific classification tasks; conversely, a comparative study by Khosa, I. [57] et al. demonstrated that a customized CNN model based on image-level thermograms (97.1% accuracy) significantly outperformed patch-level data models and traditional ML methods in DFU recognition, providing a practical reference for data selection and model design (Table 4).

**Table 4:** Performance of AI models in the grading and classification of diabetic foot ulcers

Category	Author	Technology	Core Model	Data Scale and Type	Core Performance Results
Grading based on Clinical Data	Basiri, R. [42] et al.	Machine Learning	Random Forest	890 wound assessment events from 268 patients	Mean AUC: 0.73; Accuracy: 65% (for healing stage classification)
Deep Learning - Images	Wang, Z. [43] et al.	Deep Learning	U-Net, ResNet50	1944 DFU images	Classification accuracy: 95.34%
	Wang, J.G. [44] et al.	Deep Learning	U-Net, ResNet50	1944 DFU images	Classification accuracy: 95.34%
	Li, P.L. [45] et al.	Deep Learning	DiffusionNet	3D foot scan data from 114 patients	Mean accuracy: 82.9%
	Gudivaka, R.K. [46] et al.	Machine Learning	Reinforcement Learning+CPPN + ELM	Existing DFU image datasets	Overall classification accuracy: 92.5% (six-grade classification)
	Biswas, S. [47] et al.	Deep Learning	FusionNet	6963 foot skin images	Accuracy: 99.05%; AUC: 99.09%
	Mahmud, M.I. [48] et al.	Deep Learning	EfficientNetB0 + SVM	Mixed dataset of 3000 images	Accuracy: 99.33%; AUC: 0.99
Grading/Classification based on Thermal Images	Khandakar, A. [49] et al.	Deep Learning	VGG19	167 pairs of foot thermal images	Accuracy: 95.08%; F1-score: 95.08%
	Sharma, N. [50] et al.	Deep Learning	InceptionV3	208 foot thermal images	Accuracy: 92.7%; AUC: 0.93
	Muralidhara, S. [51] et al.	Deep Learning	Custom CNN	167 plantar thermal images	6-class mean accuracy: 0.9827
	Cruz-Vega, I. [52] et al.	Deep Learning	DFTNet	110 thermal images from diabetic patients	Mean accuracy: 94.53%; Sensitivity: 95.34%
	Eldin, A.S. [53] et al.	Deep Learning	Lightweight DNN	1670 plantar thermal images	Accuracy: 98.51%; AUC: 1.00
	Bagavathiappan, S. [23] et al.	Deep Learning	ResNet50V2	153 plantar thermal images	Accuracy: 71.8%; Sensitivity: 81.2%
	Panamonta, V. [54] et al.	Deep Learning	ResNet50V2	Thermal images from 124 diabetic patients	Accuracy: 71.8%; Sensitivity: 81.2%
	Verma, G. [55] et al.	Deep Learning	EfficientNetB0	1055 foot thermal images	Accuracy: 99.4% (after preprocessing)
	Khandakar, A. [56] et al.	Machine Learning	MLP	Thermal images from 122 diabetic patients	5-fold CV accuracy: 90.1%; Weighted F1: 91.18%
	Khosa, I. [57] et al.	Deep Learning	Custom CNN	Thermal images from 167 subjects	Accuracy: 97.1%; AUC: 97.6%

## Individualized prognostic prediction: AI-driven precise assessment of outcome risk

Prognostic prediction represents a core scenario where AI demonstrates its capabilities in multi-dimensional information integration and complex pattern recognition; it is also a domain where ML models (such as XGBoost, LightGBM, and Random Forest) particularly excel. By integrating clinical features, laboratory indicators, imaging data, and comorbidity information, AI models transcend the limitations of traditional population-based statistics. They enable the individualized prediction of healing outcomes, amputation risk, long-term survival, and recurrence probability for patients with DFU. This capability provides critical decision support for selecting the timing of clinical interventions, optimizing therapeutic strategies, and ensuring the rational allocation of resources.

### Prediction of healing and refractory nature

The precise prediction of wound healing outcomes is a prerequisite for individualized DFU therapy. Related research has concentrated on simplifying predictive variables, enhancing model generalizability, and capturing the temporal characteristics of the healing process. Margolis, D.J. [58] et al., utilizing data from a multi-center prospective cohort of 207 DFU patients, re-validated the predictive value of wound area and duration. A Logistic Regression model, incorporating only these two variables, achieved an AUC of 0.7051 for predicting healing at 16 weeks. This performance was comparable to that of multivariable models, thereby confirming the enduring and robust prognostic value of these two factors.

Wang, S. [59] et al., focusing on 10 clinical features (including random blood glucose, C-reactive protein, and wound area) from 362 DFU patients, compared six ML models. The study found that the Naïve Bayes model demonstrated optimal generalization capability, achieving an AUC of 0.864 and a recall of 0.907 in predicting refractory DFU. This provides a practical tool for the early identification of patients with a poor prognosis.

In the domains of temporal prediction and the exploration of multi-dimensional influencing factors, Spinazzola, E. [60] et al. utilized follow-up data from 1,766 DFU cases. They employed a three-layer Long Short-Term Memory (LSTM) DL model to capture dynamic healing characteristics, achieving 80% accuracy and an AUC of 0.85 in predicting wound improvement or deterioration by the next follow-up visit. This study identified wound depth, area, and granulation tissue percentage as key influencing factors. Concurrently, Pereira, M.G. [61] et al. utilized a decision tree algorithm to integrate sociodemographic, clinical, biochemical, and psychological variables from 153 patients with chronic DFU. The predictive model they constructed revealed an association (F1 score > 0.7) between healing outcomes and indicators such as illness perception, Interleukin-6 (IL-6), and microRNA-146a-5p.

### Amputation risk prediction

Amputation risk prediction represents the most central focus within DFU prognostic research. This area is dominated by gradient boosting models, such as XGBoost, GBM, and LightGBM, which have been used to establish a predictive framework covering diverse amputation types and patient populations. Moreover, the application of interpretability techniques (e.g., SHAP) has further enhanced the clinical applicability of these models.

In the area of precision-stratified prediction, Gao, L. [62] et al. developed an XGBoost binary classification model based on 29 clinical variables from 599 DFU patients. This model achieved precise differentiation among “no amputation,” “minor amputation,” and “major amputation.” Notably, the model for predicting major amputation achieved an AUC of 0.977, and SHAP analysis identified key predictive factors including Wagner grade 4/5, OM, and high C-Reactive Protein (CRP) levels. Similarly, Oei, C.W. [63] et al., analyzing data from 2,559 DFU hospitalization events, developed an XGBoost model that achieved an AUC of 0.820 for predicting major amputation within 180 days. This study, also utilizing the SHAP method, elucidated the central roles of white blood cell count and the Charlson Comorbidity Index.

Multiple high-quality studies have validated the efficacy and generalizability of these models: Liu, Z. [64] et al., based on 17 key features from 598 DFU patients, constructed a GBM model that achieved an AUC of 0.9499 and an accuracy of 0.9408 in major amputation prediction, outperforming other comparator models; in a multi-center study by Tao, H. [65] et al., an XGBoost model performed excellently in both the training cohort of 1,035 patients (AUC = 0.93) and the external validation cohort of 297 patients (AUC = 0.83), confirming its robust generalization capability; Xie, P. [66] et al. employed theLightGBM algorithm to build a multi-class model, achieving a weighted-average AUC of 0 .89 among 618 DFU inpatients, which provided an interpretable tool for the refined assessment of amputation risk; Wang, S. [67] et al., focusing on 362 patients with UT Grade 3 DFU, constructed an XGBoost model that predicted minor amputation risk with an AUC of 0.881 and an accuracy of 0.814, offering a reference for interventions in this specific patient grade. Furthermore, research by Kim, R.B. [68] et al. confirmed that ML models built on image features not only can predict healing but also demonstrate potential in amputation risk assessment, providing technological support for telemedicine.

Studies from specialized perspectives have enriched the amputation prediction framework: Sánchez C.A. [69] et al., through CART analysis, found that a Wagner grade > 3 is the strongest predictor for amputation within 30 days in DFU patients (AUC = 0.764); Farideh Mostafavi, F. [70] et al. compared six IWGDF-approved wound classification systems and confirmed that DIAFORA (using an SVM-linear model, AUC = 87.0%) and the WIfI (Wound, Ischemia, and Foot Infection) system possess the optimal capability for predicting amputation; Chen, Y.L. [71] et al. discovered that different outcomes must be matched with specific models, noting that a CNN model performed best in amputation prediction (AUC = 0.939, sensitivity 92.6%).

### Prediction of mortality and major adverse cardiovascular events risk

The prognostic horizon of AI has expanded to encompass long-term survival and cardiovascular outcomes, providing technological support for the comprehensive risk management of DFU patients. This scope includes the general population, specific periods (such as the COVID-19 pandemic), and cohorts with concurrent complications.

Regarding long-term mortality risk prediction, Popa, A.D. [72] et al., based on clinical and biological data from 635 DFU patients, constructed a Multi-Layer Perceptron (MLP) neural network model. This model successfully predicted 5-year (test set AUC = 0.73) and 10-year (test set AUC = 0.7339) mortality risk. Age, ulcer grade, and renal function indicators were identified as core predictive factors, a finding of significant public health importance. Matsinhe, C. [73] et al. focused on 114 patients with diabetic foot sepsis (DFS) during the COVID-19 pandemic. The Random Forest model they constructed achieved an AUC of 0.965 and an accuracy of 0.895 in predicting mortality, identifying advanced age and COVID-19 positivity as key risk factors. Kpene, G.E. [74] et al.found, among 328 type 2 diabetes inpatients, that their decision tree model achieved 100% accuracy in predicting mortality, with nephropathy being the core predictive feature. Research by Deng, L. [75] et al. confirmed that acute hyperglycemic crisis (HCE) is an independent risk factor for mortality in DFU patients; the XGBoost model they developed achieved an AUC of 0.68 and an accuracy of 0.69 for mortality prediction.

In the prediction of MACE and cardiovascular events, Zheng, L. [76] et al. utilized data from 504 DF patients. The Random Forest model they constructed achieved an AUC of 0.70 for predicting 5-year MACE risk, significantly outperforming Logistic Regression and Support Vector Machine (SVM) models. Nabrdalik, K. [77] et al. employed the RUSBoost algorithm, based on 12 key indicators from 1,735 diabetic patients, to achieve an AUC of 0.62–0.72 for cardiovascular event prediction, effectively identifying high-risk individuals.

Prognostic research pertaining to specific periods has also advanced: Du, C., et al. [78] et al. compared data from 46 DFU patients before and after the COVID-19 lockdown. They found that the lockdown period was associated with prolonged pre-hospital delays and increased mortality rates. The XGBoost model they developed achieved an AUC of 0.94 for predicting mortality and an AUC of 0.86 for predicting amputation, providing a valuable tool for risk assessment in special scenarios, such as pandemics.

### Recurrence risk prediction

The precise prediction of DFU recurrence risk is a critical component of chronic disease management, as it facilitates the development of targeted preventive strategies. Hong, S. [79] et al., utilizing clinical data from 138 elderly diabetic patients (including age, glycemic control, and history of smoking and alcohol use), compared multiple ML models. Their findings indicated that the Support Vector Machine (SVM) model achieved an accuracy of 93% in predicting DFU recurrence, significantly outperforming other models such as XGBoost and Random Forest. This research provides a reliable tool for recurrence prevention within the elderly patient population (Table 5).

**Table 5:** Performance of AI models in predicting prognostic outcomes for diabetic foot ulcers

Prediction Target	Author	Technology	Core Model	Data Scale and Type	Core Performance Results
Healing/Non-healing	Margolis, D.J. [58] et al.	Machine Learning	Logistic Regression	207 DFU patients clinical data	16-week healing prediction AUC = 0.7051
	Wang, S. [59] et al.	Machine Learning	Naïve Bayes	362 DFU patients clinical data	AUC: 0.864; Recall: 0.907
	Spinazzola, E. [60] et al.	Deep Learning	LSTM	Case data from 1766 DFUs	Accuracy: 80%; AUC: 0.85
	Pereira, M.G. [61] et al.	Machine Learning	Decision Tree	153 chronic DFU patients data	Generated predictive model for healing (F1 > 0.7)
Amputation Risk	Gao, L. [62] et al.	Machine Learning	XGBoost	599 DFU patients data	Major amputation prediction AUC: 0.977
	Oei, C.W. [63] et al.	Machine Learning	XGBoost	2559 DFU hospitalization events	Major amputation prediction AUC: 0.820
	Liu, Z. [64] et al.	Machine Learning	GBM	598 DFU patients data	AUC: 0.9499; Accuracy: 0.9408
	Tao, H. [65] et al.	Machine Learning	XGBoost	1035 DFU patients data	Internal validation AUC: 0.93; External validation AUC: 0.83
	Xie, P. [66] et al.	Machine Learning	LightGBM	618 DFU hospitalized patients	Weighted average AUC: 0.89
	Wang, S. [67] et al.	Machine Learning	XGBoost	362 UT grade 3 DFU patients	AUC: 0.881; Accuracy: 0.814
	Kim, R.B. [68] et al.	Machine Learning	Random Forest, SVM	Clinical + image features from 113 patients	Healing prediction AUROC: 0.760–0.794 (manual image features best)
	Sánchez C.A. [69] et al.	Machine Learning	CART	553 affected feet data	30-day amputation prediction AUC: 0.764
	Mostafavi, F. [70] et al.	Machine Learning	SVM, etc.	616 DFUs from 400 patients	DIAFORA system amputation prediction AUC: 87.0%
	Chen, Y.L. [71] et al.	Deep Learning	CNN	200 DF hospitalized patients data	Amputation prediction AUC: 0.939; Sensitivity: 92.6%
Mortality/MACE Risk	Popa, A.D. [72] et al.	Machine Learning	MLP	635 DFU patients data	5-year mortality prediction test set AUC: 0.73
	Matsinhe, C. [73] et al.	Machine Learning	Random Forest	114 DFS patient records	Mortality prediction AUC: 0.965; Accuracy: 0.895
	Deng, L. [75] et al.	Machine Learning	XGBoost	120 DFU patients data	Mortality prediction AUC: 0.68; Accuracy: 0.69
	Zheng, L. [76] et al.	Machine Learning	Random Forest	504 DF patients data	MACE prediction AUC: 0.70
	Nabrdalik, K. [77] et al.	Machine Learning	RUSBoost	1735 diabetic patients data	CV event prediction AUC: 0.62–0.72
	Du, C. [78] et al.	Machine Learning	XGBoost	46 DFU hospitalized patients data	Mortality prediction AUC: 0.94; Amputation prediction AUC: 0.86
Recurrence Risk	Hong, S. [79] et al.	Machine Learning	SVM	138 elderly diabetic patients data	Accuracy: 93%

## Outlook

The prevention and treatment of DF is a complex systems engineering endeavor, involving multidisciplinary and multifaceted approaches. Although current research has achieved significant progress in diagnostic technologies and therapeutic methods, future development still faces numerous challenges and opportunities.

### The core value of nursing management and the prospects of AI empowerment

Nursing plays an indispensable role in the prevention, management, and retardation of progression for diabetic foot. Its core functions lie in high-risk population screening, personalized interventions, and continuous health self-management. However, there remains a relative deficiency in objective and standardized research specifically targeting the nursing domain. AI technology offers an innovative pathway to compensate for this limitation. Recent evidence from Ju HH et al. [80] demonstrates that nurse-led telehealth programs are highly feasible and can significantly improve patient self-care behaviors, increasing the frequency of foot self-examinations (*P*<.001) through structured remote education. Özgür, S. [81] et al., utilizing ML models such as XGBoost and LightGBM, precisely identified key factors influencing foot care self-management, including age, A1c levels, and income. They leveraged AI to process gait data, formulating personalized pressure-relief nursing plans. Expanding on this, Baseman C et al. [82] highlighted the role of computer vision (CV) and machine learning in automating the “Full Foot Exam,” enabling remote monitoring and automated classification of wound pathology with F1 scores exceeding 80%. This shift toward “Care in Place” not only improves clinical outcomes but also addresses critical health disparities; by providing accessible, AI-augmented educational tools, it is possible to reduce the disproportionate impact of DFUs on communities of color and mitigate inherent provider biases that often hinder high-quality care for marginalized populations. This approach, combined with a coordinated “hospital-community-home” management system and the potential integration of smart dressings for real-time microenvironment sensing [83], successfully reduced the annual foot ulcer recurrence rate to below 1%. This compellingly demonstrates that the deep integration of AI into the nursing management ecosystem is an inevitable trend for achieving precision prevention and control of diabetic foot.

### AI-powered intelligent advancement of imaging technologies

At present, imaging is an indispensable modality for the assessment of DFU; however, its integration with AI technology remains in a nascent stage, possessing immense potential for advancement.

#### Empowerment of foundational diagnosis based on anatomical structure

At the diagnostic level, plain X-ray radiography is frequently employed as the first-line screening tool for DFU diagnosis due to its high accessibility and low cost. It can effectively identify osseous structural changes but exhibits low sensitivity for detecting gas or foreign bodies within soft tissues [84]. CT, by virtue of its clear visualization of cortical erosion and subtle osteodystrophic changes, has become an important modality for assessing DFU-related bone destruction in emergency settings [85]. MRI, offering the advantages of being radiation-free and high-resolution, can acutely identify bone marrow edema and abscesses. Furthermore, it aids in the differentiation of OM from CNO through multi-sequence imaging techniques [86], providing critical evidence for the diagnosis of complex DFU. Although X-ray, CT, and MRI are conventional imaging modalities for the clinical diagnosis of DFU, a significant research gap currently exists within the field. Research integrating AI technologies with these traditional imaging modalities for precision DFU diagnosis and treatment remains scarce, and a mature technological framework has yet to be established. This void not only restricts the full realization of the clinical value of traditional imaging techniques but also retards the progression of DFU diagnosis toward an “objective, standardized, and efficient” paradigm. Future efforts must prioritize this direction. Through multi-center collaborations, the accumulation of high-quality annotated data is essential to provide new pathways for the intelligent upgrading of conventional imaging technologies, thereby enhancing the precision and efficiency of DFU diagnosis.

#### Bridge to endocrinology: linking AI imaging phenotypes with systemic metabolic signatures

AI-derived imaging features should be re‑conceptualized as digital biomarkers of systemic metabolic burden beyond their role in automating diagnosis. This paradigm shift directly aligns diabetic foot ulcer assessment with endocrine principles and is supported by emerging evidence from multiple imaging dimensions.

Quantitative parameters from bone SPECT/CT such as SUVmean correlate with systemic inflammatory markers including C‑reactive protein and the ESR×CRP index [87]. In diabetic foot osteomyelitis, localized bone uptake thus reflects both infection extent and the patient’s global inflammatory state, an insight that may guide anti‑inflammatory or immunomodulatory therapy. The severity and prognosis of diabetic foot infections are governed by a cytokine cascade involving TNF‑α, IL‑6, and IFN‑γ [88]; incorporating these circulating mediators into AI‑driven image analysis transforms morphological assessment into a holistic endocrine‑metabolic evaluation platform that considers local tissue damage and systemic immune dysregulation together. AI‑quantified perfusion heterogeneity on thermography correlates with HbA1c variability [89], indicating that microvascular responsiveness captured non‑invasively through thermal patterns acts as a sensitive readout of long‑term glycemic instability and links foot imaging to metabolic memory.

These cross-modal associations demonstrate that foot imaging phenotypes are compressed representations of systemic diabetic pathophysiology rather than isolated technical artifacts. Future AI models for DFU should prioritize fusing local imaging signatures with systemic metabolic indicators including advanced glycation end-products, continuous glucose monitoring metrics, and inflammatory cytokine profiles. Such integration will advance a personalized, endocrinology‑informed precision medicine framework wherein foot treatment is inseparable from host management.

Furthermore, this bridge to endocrinology extends to the precision selection of glucose-lowering therapies. Emerging research (e.g., Hong AT [90] et al. suggests that AI may support patient-specific phenotyping to inform personalized medication regimens, such as identifying potential candidates (decision support) for GLP-1 receptor agonists (GLP-1RAs) versus SGLT2 inhibitors. By analyzing multi-omics data and longitudinal clinical trajectories, AI-driven approaches can help stratify which patients may be more likely to derive cardiovascular and renal benefits from GLP-1RAs—factors that may indirectly influence distal perfusion and wound healing capacity. This predictive capability allows for a transition from a “one-size-fits-all” drug escalation to an AI-informed strategy that supports clinician decision-making and prioritizes medications with the highest potential to improve the systemic metabolic environment of the DFU patient. However, the current evidence base does not justify AI “recommending” specific drugs, and observational associations should not be interpreted as causal benefit for DFU outcomes without prospective evaluation and causal-inference safeguards (e.g., to mitigate confounding-by-indication). In practice, AI outputs should be framed as risk stratification or eligibility signals (not prescriptions), and evaluated against hard clinical endpoints such as wound closure, re-ulceration, hospitalization, and amputation-free survival.

#### Advancing ischemic DFU management and vascular surgical interventions

The management of ischemic DFU remains a significant challenge due to the complexity of lower extremity arterial disease. AI offers transformative benefits in this domain by providing objective and automated assessments of vascular patency. As demonstrated by Wu C. [26] et al., deep learning models can be trained to detect and score lower extremity arterial stenosis (LEAS) from imaging data with high precision. Such AI-driven tools can effectively filter out “noise” from vascular wall calcification and surrounding tissues, which often confound manual interpretation. In the context of vascular surgery, AI can assist in preoperative planning by precisely identifying the anatomical sites of maximum stenosis and predicting the hemodynamic success of revascularization procedures. By providing standardized Lower Extremity Arterial Stenosis Scores (LEASS), AI enables clinicians to make more informed decisions regarding the necessity and timing of surgical versus endovascular interventions, ultimately enhancing limb salvage rates in ischemic DFU patients.

### The breakthrough potential of AI in precise etiological diagnosis and antibiotic optimization

The ultimate objective of DFU treatment is to achieve healing and prevent wound infection [91]. The management of infection relies primarily on antibiotics [92]; however, the inherent delay in traditional diagnostic workflows remains a primary driver of antibiotic overuse [93] and the escalating challenge of treatment-resistant recurrences [91]. Standard clinical protocols require the collection of specimens via curettage, aspiration, or biopsy [94], but the 24–48 h delay required for culturing common pathogens—such as aerobic S. aureus, E. coli, and anaerobic Peptostreptococcus [95–97]—often forces a reliance on empirical broad-spectrum therapy. While necessary, this delay challenges the core antibiotic principles of selectivity, safety, and cost-effectiveness [98]. AI-driven technologies are now emerging to bridge this critical gap through two transformative pathways.Initial therapy should commence promptly as an empirical treatment [99], based on the severity of the infection, existing culture reports, or the local prevalence of pathogens (particularly drug-resistant strains).

#### AI-guided discovery of novel antibiotic classes

Beyond optimizing the administration of existing drugs, AI is revolutionizing the discovery of novel antibiotics specifically tailored to combat the multidrug-resistant (MDR) pathogens prevalent in IDFU, such as MRSA. As pioneered by Wong F et al. [100], explainable deep learning models can now explore vast chemical spaces to identify novel structural classes of antibiotics. By identifying specific chemical substructures associated with antimicrobial activity while minimizing human cell cytotoxicity, AI-driven platforms can accelerate the development of next-generation antibiotics that are effective against recalcitrant DFU biofilms. This move toward “AI-guided antibiotic discovery” addresses the critical shortage of effective agents against drug-resistant IDFU strains.

#### Non-invasive etiological prediction via imaging AI

In parallel, AI offers the potential to optimize initial empirical therapy by providing early etiological clues before culture results become available. Future models capable of directly analyzing MRI or CT scans could differentiate infectious pathogens—distinguishing between aerobic and anaerobic signatures—in a non-invasive and rapid manner. By utilizing DL algorithms to identify specific tissue reactions and gas distribution patterns uniquely induced by different microorganisms, AI can provide immediate diagnostic support. This capability allows clinicians to adhere to the principles of “narrow-spectrum and precise” therapy from the outset, significantly reducing the systemic burden of antibiotic overuse and ultimately improving the long-term prognosis for DFU patients.

### Selection criteria and robustness analysis of AI models

The clinical efficacy of AI in DFU management is fundamentally dependent on the robust selection of models tailored to specific data modalities and clinical objectives. As summarized in Table 6, the transition from experimental research to standardized clinical practice requires a deep understanding of each model’s strengths and limitations. For instance, while Deep Learning (DL) excels in complex image segmentation, its “black-box” nature necessitates integration with Explainable AI (XAI) to ensure safety in surgical planning. Conversely, for primary care screening where datasets are often structured but sparse, ensemble learning models like XGBoost provide superior interpretability and efficiency. Ensuring model robustness through multi-center validation and addressing data heterogeneity are essential steps for the future standardization of DFU clinical trials and nursing protocols.

**Table 6:** Comparative analysis and clinical selection of AI models identified in DFU management

Model Category	Representative Algorithms & Studies	Clinical Application in DFU	Advantages	Disadvantages
Classical ML/Ensemble Learning	XGBoost, LightGBM (Özgür [81]), Random Forest, SVM	Risk stratification; Identifying self-management factors (age, A1c); Amputation prediction (Gao [62]).	Efficient for structured data; Low computational cost; High interpretability for clinicians.	Cannot process raw images directly; Requires manual feature engineering.
Deep Learning (DL)	CNN, Mask R-CNN, YOLO (Baseman [82], Cakir [31], Wu [26])	Automated wound segmentation; OM vs. CNO differentiation; Arterial stenosis detection.	High accuracy in image recognition; Automatically extracts complex visual features.	Black-box nature (low interpretability); Requires large-scale annotated data.
Sequence Models	LSTM (Long Short-Term Memory), GRU (Baseman [82])	Predicting healing trajectories; Real-time gait monitoring; Recurrence forecasting.	Captures long-term temporal dependencies in longitudinal follow-up data.	Sensitive to missing data; High training complexity for long time-series.
Automated ML (AutoML)	AutoML (Xiaoling W [11])	Large-scale screening of high-risk DFU populations.	Reduces human bias in model selection; Accessible for non-expert clinicians.	Limited control over model architecture; High reliance on data quality.
Explainable AI (XAI)	SHAP, LIME, Grad-CAM (Biswas [47, 48])	Antibiotic decision support; Clinical trial risk factor interpretation.	Increases transparency; Provides rationale for AI decisions; Aligns with medical ethics.	May slightly decrease absolute predictive accuracy in some cases.

## Discussion

DFU, as one of the most severe complications of diabetes, presents a current status characterized by high incidence, high recurrence, and high amputation rates. This situation highlights the limitations of traditional diagnostic and therapeutic models in terms of precision, standardization, and accessibility. AI technology, through the integration of clinical data, medical imaging, and biomechanical signals, has demonstrated the potential to overcome these bottlenecks throughout the entire DFU diagnostic and treatment workflow, while concurrently facing a series of challenges that urgently need to be addressed.

AI has emerged as a transformative technology in the comprehensive management of DFU. It possesses multifaceted advantages: In the early warning stage, ML models based on clinical and biochemical data (e.g., the AutoML model by Xiaoling, W [11]. et al.) have enabled large-scale screening of high-risk DFU populations. In the diagnostic phase, DL models (e.g., the MRI DL model by Cakir, M. [31] et al.) have elevated the differentiation accuracy between OM and CNO to over 95%. In the domain of wound assessment, semantic segmentation models (e.g., the SeeWound 2 tool by Lindborg, K. [34] et al.) have achieved the simultaneous quantification of wound surface area, depth, and tissue composition. In prognostic judgment, ensemble learning models (e.g., the amputation prediction model by Gao, L. [62] et al.) can facilitate the individualized prediction of adverse outcomes. These advancements indicate that AI is driving a paradigm shift in DFU diagnosis and treatment, moving from “reactive treatment” to “proactive prevention” and from “population-based standards” to “individual-level precision.”

### Clinical use-case workflows: linking AI outputs to actionable decisions and DFU-relevant endpoints

Many AI studies in diabetic foot disease (DFD) focus on algorithmic performance; however, clinical adoption requires a clear mapping from model inputs and outputs to bedside actions and clinically meaningful endpoints. To enhance interpretability for non–AI-trained clinicians and demonstrate “individual-level precision,” we synthesize representative evidence from the included literature into three patient-level workflows (Tables 7, 8 and 9). These workflows are illustrative pathways (not individual case reports) intended to show how AI tools may be integrated into routine clinical processes as decision support.

**Table 7:** Clinical decision-support framework for AI-assisted diabetic foot ulcer management: from clinical questions to actionable endpoints

Clinical Question	Inputs Required	AI Task & Output	Change in Clinical Management	Relevant Clinical Endpoint(s)	Key Supporting Studies
1. How is the ulcer changing over time, and is the wound bed improving?	Standardized wound photographs (clinic or smartphone); optional metadata (date/time, location)	Automated wound localization and segmentation; quantitative morphology (area, perimeter, width/length); wound bed tissue characterization (e.g., slough/necrosis)	Enables consistent longitudinal tracking; supports escalation decisions when improvement plateaus; improves communication and documentation	Surrogate-to-endpoint linkage: Change in wound size/composition as objective monitoring signals toward wound closure/time-to-heal	[34] (SeeWound© 2 vs. digital planimetry)
2. Can we quantify depth and surface area accurately to standardize assessment?	Photographs ± depth probe measurement; in vitro models + patient wounds (including DFU)	Accuracy/precision estimates for area and depth measurement; comparison to conventional planimetry/probing	Supports standardization across sites and trials; reduces inter-rater variability; allows consistent thresholds for “response vs. non-response”	Objective measurement supports defining/monitoring healing trajectories; facilitates consistent reporting in studies	[34]
3. What tissue components are present, and can we stratify severity (e.g., Wagner grade) from images?	DFU images; manual annotations for periwound erythema, ulcer boundary, and tissues (granulation/necrosis/tendon/bone/gangrene)	Fine-grained instance segmentation of ulcer boundary + tissue components; classification of severity (e.g., Wagner grades)	Adds interpretable imaging features for triage and follow-up; highlights concerning tissues (e.g., exposed tendon/bone) for early referral/imaging	Clinically meaningful stratification (severity) and monitoring signals linked to healing risk; supports earlier intervention	[35] (Mask2former; tissue IoU; Wagner-grade classification)
4. How can remote monitoring be operationalized as a clinician–patient workflow?	Patient smartphone images uploaded to cloud; basic identifiers/time stamps; clinician portal	End-to-end wound management system: segmentation via cGAN; automated morphological metrics; longitudinal plots in clinician portal	Enables remote follow-up, trend visualization, and documentation; supports timely treatment adjustment	Supports monitoring toward wound closure and prevents delays in escalation (process endpoint)	[36] (AFSegGAN-based WMS)
5. Can a single platform support localization, phenotyping, and measurement to guide next steps?	Smartphone wound images	Detection/localization (e.g., YOLO-based), classification (infection/ischemia), and measurement; basic tissue color/texture analysis	Supports triage (e.g., suspected infection/ischemia → prompt in-person assessment); supports follow-up scheduling	Early detection of complications; indirectly supports amputation-risk reduction and timely wound closure	[37] (DFUCare platform)

**Table 8:** Infrared thermography and wearable sensors for diabetic foot risk stratification and monitoring

Clinical Question (Patient-Level)	Inputs Required (Setting)	AI Task & Output	How It Changes Management (Actionable Step)	DFU-Relevant Clinical Endpoint(s)	Key Supporting Studies
Can we identify patients at higher risk before overt ulceration, using temperature patterns?	Plantar foot infrared thermograms (clinic or community screening); optional demographics	Feature extraction from thermograms (e.g., vascular-territory temperatures, TCI, histogram-based features); classification of diabetes vs. control/abnormal thermal pattern	Flags individuals for foot-care pathway: neuropathy workup, podiatry referral, footwear counseling, closer surveillance	Earlier risk detection → aims to reduce progression to DFU (preventive endpoint)	[24] (thermogram feature ranking + ML classifiers; comparison vs. 2D CNN); #29 (thermogram-derived indices such as TCI and vascular-territory features)
Is higher plantar temperature physiologically linked to neuropathy severity (supporting clinical interpretability)?	Infrared thermography + neuropathy assessment (e.g., biothesiometry/VPT)	Correlation analysis: mean foot temperature and great-toe temperature vs. VPT-defined neuropathy status	Provides interpretable rationale for thermal monitoring; supports integrating thermography into neuropathy assessment and follow-up intensity	Risk stratification supporting ulcer prevention through earlier offloading/education	[23] (VPT > 20 V defined neuropathy; higher foot temperature; correlation with VPT; no correlation with HbA1c)
Can low-complexity sensors enable home/clinic monitoring for neuropathy-related risk (beyond imaging alone)?	Plantar pressure + temperature time-series from multi-channel sensors (wearable/clinic device)	ML classification (feature selection + k-NN) to distinguish neuropathy vs. healthy; embedded/low-resource implementation	Enables scalable screening/monitoring; triggers preventive interventions (offloading, footwear modification, education) and earlier review when risk rises	Preventive endpoint: reduce first ulcer/re-ulceration risk; supports monitoring adherence to offloading	[18] (pressure + temperature signals; high diagnostic performance; low-resource hardware feasibility)
How can thermography be operationalized in longitudinal monitoring rather than one-off screening?	Serial thermograms (single or both feet) with time stamps	Trend monitoring using interpretable thermal features (e.g., TCI/NTR/HSE or top-ranked feature sets), with validated cross-validation protocols	Allows escalation when temperature asymmetry/pattern worsens; supports remote follow-up scheduling and patient engagement	Process-to-endpoint linkage: earlier detection of deterioration → supports timely care to prevent DFU complications	[24] (feature optimization; model selection; smartphone-deployable models); #29 (feature frameworks such as TCI/NTR/HSE enabling interpretable monitoring)

**Table 9:** Advanced imaging and wound photography for differential diagnosis and telemedicine in diabetic foot complications

Clinical Question (Patient-Level)	Inputs Required	AI Task & Output	How It Changes Management (Actionable Step)	DFU-Relevant Clinical Endpoint(s)	Key Supporting Studies
In a “hot foot” with bone marrow signal abnormality (BMSA) on MRI, is this osteomyelitis (OM) or active Charcot neuro-osteoarthropathy (CNO)?	Foot MRI with standard DF protocol (T1, fat-sat T2, STIR ± post-contrast when available)	DL classification of ROI-level BMSA patterns on T1/T2 into OM vs. CNO vs. trauma-related edema; per-class sensitivity/specificity; supports one-vs-rest binary comparisons	Helps reduce diagnostic ambiguity and avoid “wrong-pathway” treatment (e.g., unnecessary prolonged antibiotics/surgery vs. delayed immobilization/offloading)	Earlier correct pathwaying → supports infection control, reduces progression, and aims to improve limb preservation (amputation-risk mitigation)	[31] (retrospective cohort; radiologist-defined ROI segmentation; ResNet-50/EfficientNet-b0 high multi-class accuracy; CNO clinically/lab defined and treatment-responsive)
How is the ground truth established for OM vs. CNO in imaging-AI studies, and what are the limitations?	Histopathology (when available), clinical/lab diagnosis + treatment response, and expert radiology ROI labeling	Transparent labeling schema: OM confirmed histopathologically; CNO defined clinically/lab and conservative-treatment response; ROIs segmented by experienced MSK radiologists using semi-automated tools	Enables clinicians to interpret outputs in context (e.g., understand that CNO is primarily a clinical diagnosis; imaging AI provides supportive evidence rather than a definitive label)	Improves trust and supports safer implementation; reduces over-reliance on model outputs	[31] (OM histopathology; CNO clinical/lab + follow-up response; ROI segmentation consensus)
From routine wound images, can we rapidly phenotype infection and ischemia to trigger timely escalation?	Smartphone wound images (remote or clinic)	Computer-vision pipeline for wound localization + binary classification of infection and ischemia; ancillary color/texture features and size measurement	Supports triage and pathwaying: suspected infection → prompt in-person assessment/cultures/antibiotics; suspected ischemia → vascular work-up/referral; supports scheduling urgency	Timely escalation supports infection control, revascularization timing, and may improve wound closure and amputation-free outcomes	[37] (DFUCare: localization + infection/ischemia classification; clinical testing with low error rates)
Can AI support telemedicine by combining phenotyping with documentation and longitudinal follow-up?	Serial wound images + basic metadata (time, location)	Platform-level integration (localization/classification/measurement) enabling longitudinal review	Earlier detection of deterioration; supports follow-up cadence adjustment and documentation	Process endpoint linked to hard outcomes: fewer delays in escalation; potential reduction in severe complications	[37] (platform workflow and clinical testing)

#### Wound quantification and healing-oriented monitoring

A near-term opportunity for AI in DFD is objective, repeatable wound assessment that reduces inter-rater variability and enables standardized documentation across clinicians and sites. AI-enabled systems can automate wound localization/segmentation and provide quantitative morphology (e.g., surface area, perimeter, width/length), and in some studies, wound bed characterization (e.g., slough/necrosis) and depth estimation. These outputs can be incorporated into longitudinal follow-up to identify non-responders (e.g., plateau in size reduction or unfavorable tissue evolution), prompting actionable steps such as escalation of therapy, modification of offloading strategy, or earlier in-person review. While wound size and tissue composition are intermediate markers rather than hard endpoints, they can be operationalized as clinically interpretable monitoring signals aligned with DFU-relevant endpoints such as complete re-epithelialization (wound closure) and time-to-heal, provided that prospective validation confirms robust endpoint linkage.

#### Thermal/temperature-based risk identification and longitudinal monitoring

Thermal imaging and temperature-related sensing offer a clinician-friendly route to early risk identification and monitoring because outputs are intuitively interpretable (e.g., abnormal temperature patterns, asymmetry, localized “hot spots”). Current evidence in this review primarily supports thermal AI as a tool for risk identification and monitoring signals, including detection of abnormal plantar temperature distributions associated with neuropathy-related risk and deployable workflows using infrared thermography or low-complexity sensor systems. In practice, these outputs can trigger preventive actions—neuropathy assessment, podiatry referral, footwear counseling, reinforcement of offloading, and closer follow-up—aiming to reduce progression toward ulceration or re-ulceration. However, most published studies have not yet established strong prospective links between thermal AI outputs and hard clinical endpoints (e.g., incident ulceration, re-ulceration, amputation-free survival). Therefore, thermal AI should currently be positioned as supportive within prevention and monitoring programs, with emphasis on standardized acquisition protocols and endpoint-driven prospective validation.

#### Diagnostic pathwaying for OM vs. CNO and phenotyping infection/ischemia

Clinical outcomes can be compromised when DFD phenotypes are misclassified—for example, when imaging and clinical features overlap between OM and CNO, or when infection/ischemia is not recognized early. Evidence summarized in this review shows that AI can assist in differentiating OM vs. CNO (and trauma-related marrow edema) on MRI using expert-defined regions of interest and reference standards that may include histopathology for OM and clinically/laboratory defined CNO with treatment-response follow-up. In parallel, smartphone-based computer-vision platforms can phenotype infection and ischemia from wound images to support triage and escalation pathways (e.g., urgent in-person assessment, antimicrobial work-up, or vascular referral). Importantly, CNO remains a clinical diagnosis; imaging AI should be framed as an adjunct providing objective supportive information, interpreted alongside history, examination, laboratory markers, and treatment response. The clinical value of these models should ultimately be judged not only by accuracy, but also by whether they reduce wrong-pathway decisions and improve DFU-relevant outcomes including timely infection control, appropriate revascularization, wound healing, and limb preservation.

#### Cross-cutting considerations for endpoint alignment and clinical translation

Across the above workflows, two principles should guide future research and implementation. First, AI-derived endpoints should be anchored to clinically meaningful outcomes rather than isolated technical metrics. For example, segmentation quality should be evaluated in relation to whether derived trajectories improve prediction of wound closure or time-to-heal; risk stratification should be assessed against incident ulceration or recurrence; and diagnostic decision support should be evaluated by timeliness and appropriateness of pathway decisions and downstream limb outcomes. Second, models should be tested under real-world constraints—heterogeneous image quality, variable acquisition protocols, different devices/sites, and evolving clinical pathways—using robust external validation and, ideally, prospective studies.

In summary, the patient-level workflows in Tables 7, 8, 9 demonstrate how AI can move from model-centric performance reporting toward clinician-actionable tools by explicitly defining inputs, outputs, clinical actions, and DFU-relevant endpoints. This translational framing may facilitate more standardized evaluation, improve clinician trust, and accelerate responsible adoption in DFD care.

### Persistent challenges in clinical translation

Despite these promising prospects, the clinical translation of AI in the DFU domain still faces critical challenges. First, model generalization capability is insufficient, as most studies are constructed based on single-center data and lack multi-center external validation 101. Second, the AI decision-making process suffers from a lack of interpretability, which affects clinician comprehension and trust. As highlighted in Table 6, the selection of AI models must prioritize robustness and clinical fit over absolute accuracy. Third, the acquisition and annotation of multi-modal data lack uniform standards, constraining large-scale model training.

### Future directions for research and implementation

To address these challenges, future efforts should focus on the following directions: constructing multi-center, standardized DFU multi-modal databases (such as the Zivot dataset by Basiri, R. [41] et al.) to provide a data foundation for improving model generalization; introducing explainable AI (XAI) techniques (e.g., Biswas, S., Mahmud, M.I. [47, 48]) to enhance clinical trust; and developing unified standards for data acquisition and annotation. At the level of clinical translation, lightweight AI tools suitable for primary care settings should be developed (e.g., thermal imaging analysis models deployable on smartphones [24], and the fusion of local foot imaging with systemic indicators (e.g., body composition from abdominal CT) should be explored to construct a more comprehensive DFU assessment system.

## Data Availability

No datasets were generated or analysed during the current study.
